# Impact of histone H4K16 acetylation on the meiotic recombination checkpoint in *Saccharomyces cerevisiae*

**DOI:** 10.15698/mic2016.12.548

**Published:** 2016-12-04

**Authors:** Santiago Cavero, Esther Herruzo, David Ontoso, Pedro A. San-Segundo

**Affiliations:** 1 Instituto de Biología Funcional y Genómica. Consejo Superior de Investigaciones Científicas and University of Salamanca, 37007 Salamanca, Spain.; 2 Present address: Department of Experimental and Health Sciences, Pompeu Fabra University, 08003-Barcelona, Spain.; 3 Present address: Molecular Biology Program, Memorial Sloan Kettering Cancer Center, New York, New York 10065, USA.

**Keywords:** meiosis, checkpoint, histone H4K16, chromatin modifications, Sir2, Pch2, Sas2

## Abstract

In meiotic cells, the pachytene checkpoint or meiotic recombination checkpoint is a surveillance mechanism that monitors critical processes, such as recombination and chromosome synapsis, which are essential for proper distribution of chromosomes to the meiotic progeny. Failures in these processes lead to the formation of aneuploid gametes. Meiotic recombination occurs in the context of chromatin; in fact, the histone methyltransferase Dot1 and the histone deacetylase Sir2 are known regulators of the pachytene checkpoint in *Saccharomyces cerevisiae*. We report here that Sas2-mediated acetylation of histone H4 at lysine 16 (H4K16ac), one of the Sir2 targets, modulates meiotic checkpoint activity in response to synaptonemal complex defects. We show that, like *sir2*, the *H4-K16Q* mutation, mimicking constitutive acetylation of H4K16, eliminates the delay in meiotic cell cycle progression imposed by the checkpoint in the synapsis-defective *zip1* mutant. We also demonstrate that, like in *dot1*, *zip1*-induced phosphorylation of the Hop1 checkpoint adaptor at threonine 318 and the ensuing Mek1 activation are impaired in *H4-K16* mutants. However, in contrast to *sir2 *and *dot1*, the *H4-K16R* and *H4-K16Q* mutations have only a minor effect in checkpoint activation and localization of the nucleolar Pch2 checkpoint factor in *ndt80*-prophase-arrested cells. We also provide evidence for a cross-talk between Dot1-dependent H3K79 methylation and H4K16ac and show that Sir2 excludes H4K16ac from the rDNA region on meiotic chromosomes. Our results reveal that proper levels of H4K16ac orchestrate this meiotic quality control mechanism and that Sir2 impinges on additional targets to fully activate the checkpoint.

## INTRODUCTION

Meiosis is a specialized type of cell division in which a single round of DNA replication is followed by two consecutive rounds of nuclear division (meiosis I and II), allowing the generation of haploid gametes from diploid progenitor cells 1,2. In the first meiotic division the segregation of homologous chromosomes (homologs) takes place, whereas during meiosis II sister chromatids separate one from each other.

Between DNA duplication and the first meiotic division, a complex series of events involving homologous chromosomes occur during the so-called meiotic prophase; namely, genetic recombination initiated by Spo11-induced DNA double-strand breaks (DSBs) 3, alignment of parental chromosomes (pairing) and tight association of homologs (synapsis) in the context of the synaptonemal complex (SC) 1,4. The SC is a highly conserved meiosis-specific tripartite structure that assembles along the lengths of paired homologous chromosomes. It consists of a central region, in which the *S. cerevisiae* Zip1 protein is the major component 5,6, and two lateral elements composed of the Hop1 and Red1 proteins. Problems in the recombinational repair of meiotic DSBs as well as defects in pairing and synapsis of homologs are situations that trigger the activation of a meiosis-specific surveillance mechanism, the so-called pachytene checkpoint or meiotic recombination checkpoint, that prevents meiotic nuclear division until those crucial processes have been completed 7,8,9. In the yeast *Saccharomyces cerevisiae*, the activation of this evolutionarily-conserved pathway by unrepaired meiotic DSBs relies on the same sensor proteins that the canonical DNA damage checkpoint operating in vegetative growing cells, specifically the Mec1 and Tel1 kinases (the yeast homologs of mammalian DNA damage sensor kinases ATR and ATM), Rad24 and the 9-1-1 complex 10,11,12,13,14. In addition, meiosis-specific proteins, present in the chromosomal axis, such as Red1 and Hop1 15,16,17, act as adaptors sustaining the activation and hyperphosphorylation of the meiosis-specific downstream effector kinase Mek1 18,19,20,21,22,23. The delay in the exit from meiotic prophase in *S. cerevisiae* is imposed predominantly by controlling the expression and localization of the meiosis-specific transcription factor Ndt80, which in turn promotes the activation of the majority of genes required for late meiotic development, including B-type cyclins and the polo-like kinase Cdc5 18,24,25,26,27, as well as by inhibiting the major cyclin-dependent kinase (CDK) Cdc28 through its Swe1-dependent phosphorylation 28,29. Budding yeast meiotic mutants such as *zip1*, defective in SC and crossover formation that leads to the accumulation of recombination intermediates 5,30,31, are invaluable genetic tools to activate and study the pachytene checkpoint.

Meiotic recombination and the checkpoint response occur in the context of chromatin, which is subject to a wide variety of histone post-translational modifications (PTMs). These histone PTMs include acetylation, methylation, phosphorylation or ubiquitylation and exert their functions either influencing the overall structure of chromatin or regulating the binding of effector molecules. Histone PTMs have important roles in transcription, replication, repair, establishment of euchromatin/heterochromatin and other aspects of eukaryotic chromosome dynamics. Various histone PTMs have been described to be involved in crucial meiotic processes, such as recombination and the pachytene checkpoint 8,9,32. In particular, it has been proposed that H3K4 trimethylation promotes the formation of Spo11-dependent meiotic DSBs in *S. cerevisiae* mediated by the tethering of the Ssp1 subunit of the Set1 complex to chromosome axes 33,34,35. Nevertheless, further mechanistic studies are required to confirm this model. In addition, previous reports have also revealed the requirement of Dot1 and Sir2 for the meiotic block triggered by the pachytene checkpoint in *zip1* mutants lacking a component of the SC 21,36,37. Dot1 is the methyltransferase required for H3K79 methylation (H3K79me), whereas Sir2 is a histone deacetylase that establishes and maintains silencing within yeast heterochromatic-like regions at telomeres, ribosomal DNA (rDNA) and silenced mating-type loci, and whose preferred histone substrates are H3K56ac and H4K16ac 38,39,40,41,42. However, in some cases, the precise meiotic role of those epigenetic modiﬁcations is not well known yet.

In this work we have investigated the role of the acetylation of lysine 16 in histone H4 (H4K16ac) during meiosis and its regulation by Sas2 and Sir2. We demonstrate that global acetylation of H4K16 does not change in either unperturbed or challenged meiosis and found that proper H4K16ac is dispensable during normal meiotic divisions. However, it is required for meiotic checkpoint activity, as manifested by the effect of *H4-K16R* and *H4-K16Q* mutants on suppression of the checkpoint-induced meiotic delay of *zip1*. These mutants show a reduction in the activity of the Mek1 meiotic effector kinase, which is most probably due to impaired Hop1 phosphorylation at threonine 318. Our results also indicate that the effect of *H4-K16R* and *H4-K16Q* mutations on the meiotic checkpoint is exerted, at least in part, through a cross-talk between H4K16ac and H3K79me. We provide cytological evidence showing that Pch2 localization is slightly altered in the H4K16ac mutants and, finally, we unveil the meiotic chromosomal distribution of H4K16ac, which is excluded from the rDNA region in a Sir2-dependent manner.

## RESULTS AND DISCUSSION

### Global levels of H4K16ac do not change in either normal or challenged meiosis

In budding yeast, the lysine 16 of histone H4 (hereafter H4K16) is primarily acetylated by Sas2, a member of the MYST-type family of histone acetyltransferases (HATs) 43,44,45,46,47 and secondarily by the essential HAT Esa1 48,49. In turn, at least* in vitro*, H4K16ac is the preferred substrate, but not the only one, of the NAD^+^-dependent Sir2 deacetylase 40,44,50,51,52. Importantly, disruption of *SIR2* leads to H4K16 hyperacetylation exclusively in heterochromatic-like regions, such as subtelomeric sequences, the rDNA locus and the silenced mating-type loci, but does not affect genome-wide H4K16ac 53. In fact, Sir2-dependent deacetylation of H4K16ac is a characteristic feature of silenced chromatin at those particular genomic domains 54. Since Sir2 has been shown to play a crucial role in the meiotic recombination checkpoint 36, we sought to explore the possible role of H4K16ac in this process.

**Figure 1:**
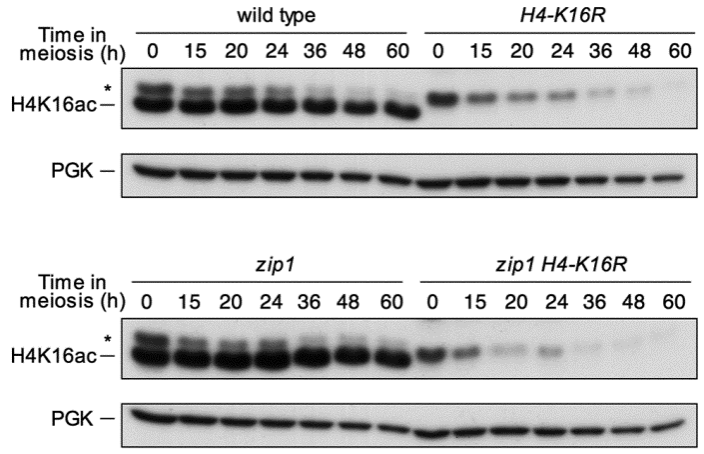
FIGURE 1: H4K16 acetylation remains unaltered during both normal and perturbed meiosis. Western blot analysis of H4K16 acetylation throughout meiosis in wild-type (DP421) and *zip1* (DP422) cells. The *H4-K16R* (DP994) and *zip1 H4-K16R* (DP995) mutant strains were used as controls for antibody specificity. PGK was used as a loading control. Asterisks mark a non-specific band.

To study the kinetics of H4K16ac accumulation during meiosis, we performed meiotic time courses as described in Materials and Methods and followed this histone mark by immunoblotting with an anti-H4K16ac antibody. A non-acetylatable *H4-K16R* mutant was used as a control for antibody specificity (Figure 1). In this preliminary approach to determine variations of this histone modification, we found that global levels of H4K16ac do not significantly change upon meiosis induction (compare time 0 with the remaining times) or during the whole length of the meiotic program (Figure 1, upper panels). Next, we wanted to determine if H4K16ac was affected by the activation of the meiotic recombination checkpoint; thus, we analyzed a *zip1* mutant, which triggers the checkpoint. We found that H4K16ac levels were also unaltered during the meiotic time courses in the *zip1* mutant (Fig. 1, lower panels), indicating that despite the role of Sir2 in the checkpoint, global levels of H4K16ac remain fairly constant when synapsis defects exist.

Previous studies have shown that histone acetylation levels, including those of H4K16, dramatically increase during the induction of an HO-induced DSB lesion and decrease during the subsequent homologous recombinational repair, presumably due to the coordinated action of histone modifying enzymes, such as Esa1 and Sir2, that are recruited to the lesion 55. This ability to modify the levels of histone acetylation is essential to maintain cell viability after exposure to DNA damaging agents or during DNA repair by homologous recombination, either because changes in histone acetylation are necessary for the recruitment of DNA repair enzymes and/or chromatin remodelers, or because they are important in downstream signaling. In fact, different H3 and H4 lysines are found acetylated upon DNA damage in yeast 56,57. Meiosis involves the generation and subsequent repair of multiple DSBs across the genome and signal transduction in the meiotic checkpoint pathway shares many components with the mitotic DNA damage checkpoint 8. However, in this study, we show that global levels of H4K16ac do not change either with the induction of the meiotic program or when meiotic chromosome synapsis defects exist (Figure 1). Nevertheless, the precise meiotic errors (incomplete recombination, chromosome structural defects or both) triggering the checkpoint in the *zip1* mutant remain to be established. In addition, in contrast to the situation in mitotic cells, meiotic DSB repair occurs in the special context of the SC with probably different chromatin modifications requirements. Moreover, in our study we have measured global levels of H4K16 acetylation and we cannot rule out the possibility that local modifications of H4K16 acetylation may occur at particular genomic regions.

### H4K16 normal acetylation is required for efficient meiotic checkpoint regulation

**Figure 2:**
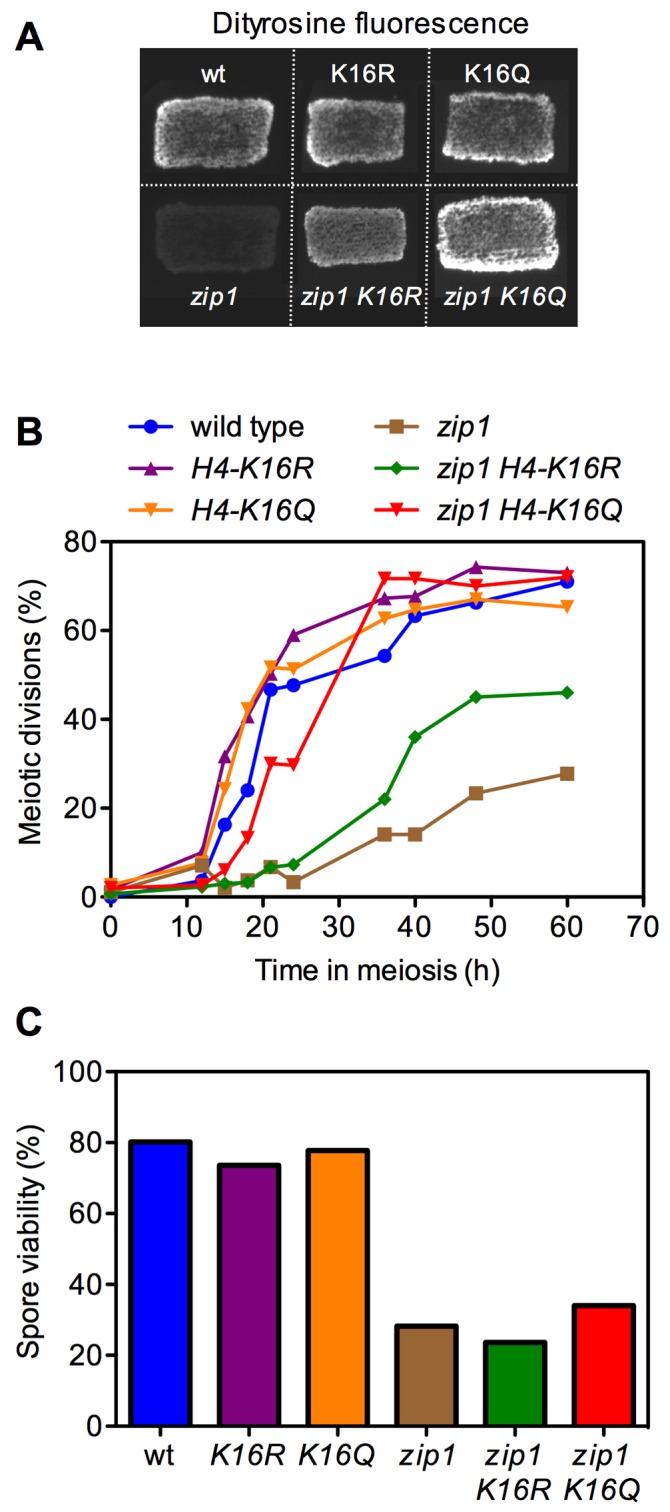
FIGURE 2: The meiotic recombination checkpoint is impaired in *H4-K16R* and *H4-K16Q* mutants. **(A)** Dityrosine fluorescence, as an indicator of sporulation, was examined after 3 days of sporulation on plates. **(B)** Time course of meiotic nuclear divisions; the percentage of cells containing two or more nuclei is represented. **(C)** Spore viability, as assessed by asci dissection, is presented. At least 144 spores were scored for each strain. Strains used in (A) are DP421 (wild type), DP994 (*H4-K16R*), DP1000 (*H4-K16Q*), DP422 (*zip1*), DP995 (*zip1 H4-K16R*) and DP1001 (*zip1 H4-K16Q*)*.* Strains used in (B) and (C) are DP634 (wild type), DP635 (*H4-K16R)*, DP636 (*H4-K16Q)*, DP639 (*zip1)*, DP640 (*zip1 H4-K16R)* and DP641 (*zip1 H4-K16Q).*

To further investigate the role of H4K16ac in meiosis, several meiotic events were analyzed in *H4-K16R* (non-acetylatable) and *H4-K16Q* (mimicking constitutive acetylation) mutants, both in a wild-type (unperturbed meiosis) and a *zip1* background (triggering meiotic checkpoint activation). The kinetics of meiotic nuclear divisions was monitored by DAPI staining of nuclei. Dityrosine fluorescence, a specific component of mature spores, was used as a semi-quantitative indicator for sporulation efficiency. Finally, spore viability that reflects the fidelity of meiotic chromosome segregation and the integrity of the spore genome was determined by tetrad dissection. In an otherwise wild-type background, the *H4-K16R *and* H4-K16Q* single mutants showed no or little meiotic defects (Figure 2). The progression through meiosis was normal (Figure 2B, S1A) and resulted in the formation of mature dityrosine-containing spores (Figure 2A) with a high viability similar to that of the wild type (Figure 2C). These observations suggest that normal regulation of H4K16ac is dispensable in unperturbed meiosis.

As previously described, the *zip1* mutant, where the pachytene checkpoint is triggered, showed a strong delay in meiotic progression and the formation of mature spores was dramatically reduced (Figure 2A, 2B, S1A). Notably, the *H4-K16R* and *H4-K16Q* mutations were able to partially (*K16R*) or completely (*K16Q*) alleviate the checkpoint-dependent meiotic block: the *zip1 H4-K16Q *and* zip1 H4-K16R *double mutants progressed faster into meiosis (Figure 2B, S1A) and formed dityrosine-containing spores in a higher proportion than *zip1* cells (Figure 2A); however, spore viability remained low (Figure 2C) indicating that although *zip1 H4-K16Q *and* zip1 H4-K16R *cells were able to progress into meiosis and to form mature spores, the problems caused by the lack of Zip1 persist. Thus, the status of H4K16ac modulates meiotic progression in the *zip1* mutant. Interestingly, the *H4-K16Q* mutant mimicking constitutive acetylation shows a stronger checkpoint defect, similar to the lack of the Sir2 deacetylase 36 (see below).

### *H4-K16R* and *H4-K16Q* mutants are defective in the maintenance, but not the establishment, of checkpoint-induced Mek1 activation

**Figure 3:**
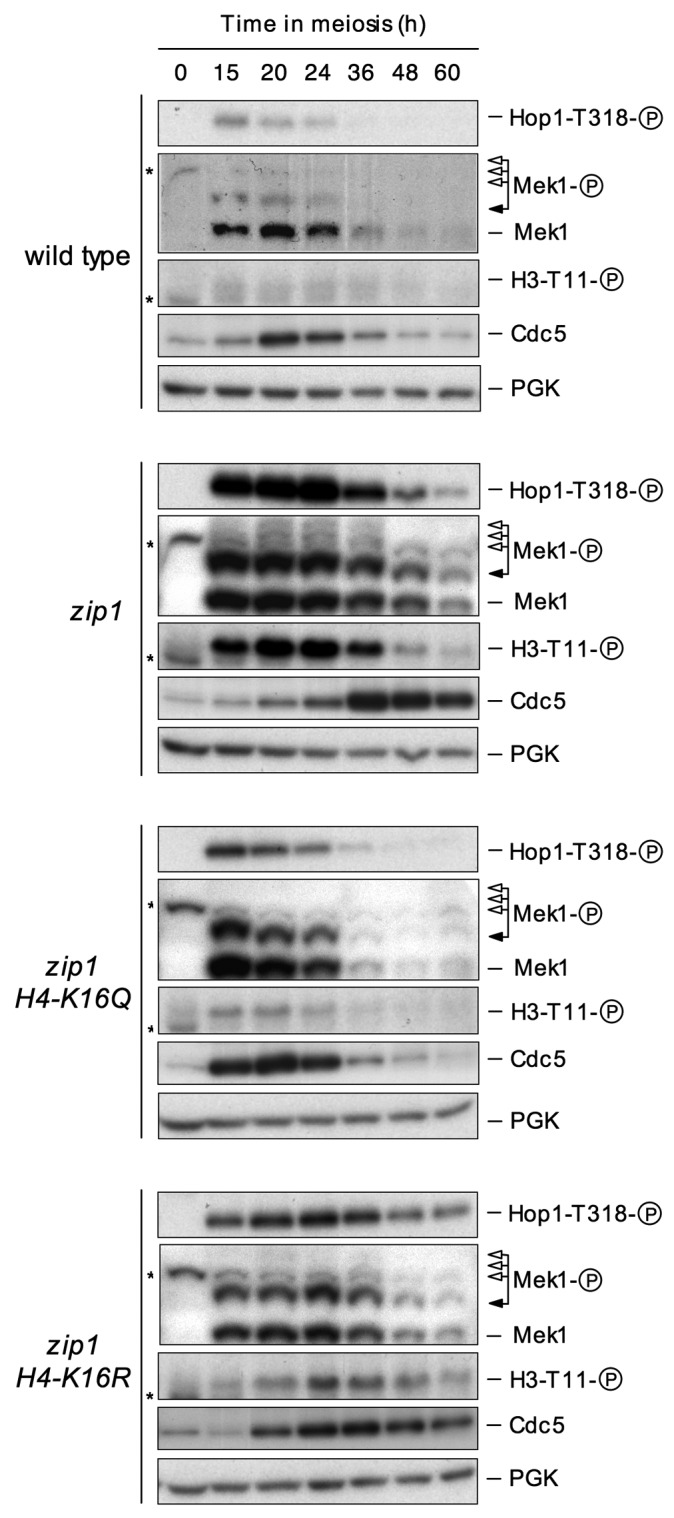
FIGURE 3: H4K16 acetylation is necessary for normal Mek1 and Hop1 phosphorylation. Western blot analysis of Mek1 and Hop1 activation in wild type (DP421), *zip1* (DP422), *zip1 H4-K16Q* (DP1001) and *zip1 H4-K16R* (DP995) strains throughout meiosis. Black arrows point the Mec1/Tel1-dependent phosphorylated form of Mek1, whereas white arrows mark the bands resulting from Mek1 autophosphorylation 21. Asterisks mark non-specific bands. H3T11 phosphorylation and Cdc5 inhibition were used as additional molecular markers for checkpoint activation. PGK was used as a loading control.

To investigate the meiotic checkpoint role of H4K16ac more directly at a molecular level, we followed the status of Mek1 activation throughout meiotic time courses in the *zip1 H4-K16R* and *zip1 H4-K16Q* mutants using high-resolution Phos-tag gels. The appearance of hyper-phosphorylated Mek1 isoforms is indicative of meiotic checkpoint activation 21. The threonine 11 of histone 3 has been identified as one of Mek1 downstream targets 58. Although the role of H3T11ph in meiosis, if any, is still unclear, it is a useful additional reporter for Mek1 kinase activity (Figure 3) 59. In wild-type cells, Mek1 levels rose transiently during meiotic prophase (peak at 20 hours) and then progressively declined as meiosis I and II and sporulation took place. Phosphorylated forms of Mek1 and H3T11ph remained at very low levels during the whole meiotic time course (Figure 3, upper panel). In contrast, robust Mek1 activation, as shown by the appearance of additional slow migrating and stronger phosphorylated Mek1 forms, and marked H3T11ph could be detected in the *zip1* mutant (Figure 3, second panel), consistent with its pronounced meiotic delay triggered by the checkpoint (Figure 2B). We next examined the *zip1 H4-K16R* and *zip1 H4-K16Q* double mutants. Remarkably, according with the complete suppression of the meiotic delay (Figure 2B), Mek1 activation was severely impaired in the *zip1 H4-K16Q* double mutant, as manifested by the absence of the upper Mek1 phosphorylated forms and low levels of H3T11ph (Figure 3, third panel). The *zip1 H4-K16R*, which shows only a partial checkpoint defect (Figure 2B), showed a milder reduction in both the levels and the duration of Mek1 activation and H3T11ph (Figure 3, bottom panels).

Mec1/Tel1-dependent phosphorylation of Hop1 at defined S/T-Q sites is required for Mek1 hyperphosphorylation and activation, as well as for meiotic checkpoint activity 15. Among the several S/T-Q sites targeted by Mec1/Tel1 in Hop1, phosphorylation of threonine 318 together with phosphorylation of serine 298 are crucial events in the meiotic checkpoint network to coordinate recombination and meiotic progression 60. We examined the levels of Hop1-T318 phosphorylation throughout the meiotic time courses using a phospho-specific antibody as an upstream marker for *zip1*-induced checkpoint activation 59. During normal meiosis, only a very weak and transient Hop1-T318ph signal could be detected during the meiotic prophase, coinciding with the weak activation observed in Mek1 (Figure 3, upper panel). However, in *zip1* mutant cells triggering the activation of the pachytene checkpoint, Hop1-T318ph dramatically increased (Figure 3, second panel). We next analyzed the *zip1 H4-K16R* and *zip1 H4-K16Q* double mutants and we found a reduction in Hop1-T318 phosphorylation, very similar to that observed in Mek1 activity (Figure 3, third and bottom panels). Again, the effect of *H4-K16Q* was much stronger.

To further support the results shown above, we also analyzed a downstream target of the meiotic recombination checkpoint, the Cdc5 polo-like kinase. Cdc5 is one of the most prominent members of a large set of genes under the control of the meiosis-specific Ndt80 transcription factor, with a number of functions in meiosis including the exit from pachytene and entry into the first meiotic division 18,24,61,62,63,64. In wild-type cells, low levels of Cdc5 were detected in vegetative cell cycle, prior to entering meiosis; those levels peaked during mid-meiosis and then declined. Meanwhile, in a *zip1* mutant the production of Cdc5 was clearly delayed (Figure 3, top and second panels), according with the slower meiotic progression (Figure 2B). In contrast, earlier induction of Cdc5 production was completely or partially restored in the *zip1 H4-K16Q* and *zip1 H4-K16R* double mutants, respectively (Figure 3, third and bottom panels), which is again consistent with the meiotic progression of these mutants.

All together, these results confirm the effect of *H4-K16Q* and *H4-K16R* mutations in meiotic progression and indicate that the checkpoint defects observed most probably arise from the failure to efficiently phosphorylate Hop1 and Mek1. Thus, H4K16ac is required for both Hop1 phosphorylation and the ensuing Mek1 activation in the meiotic recombination checkpoint pathway.

Interestingly, the substitution of the lysine 16 of histone 4 with differently charged residues resulted in slightly different outcomes. Similar to the lack of the Sir2 deacetylase 36 the *H4-K16Q* substitution, mimicking the constitutively acetylated state of lysine, completely abolished the meiotic block imposed by *ZIP1* disruption, as well as the phosphorylation of Mek1 and Hop1. Conversely, substitution of the lysine by arginine, a residue that cannot be acetylated, *H4-K16R*, only showed a partial effect on the meiotic progression as well as in the Hop1 and Mek1 phosphorylation (Figures 2 and 3). Curiously, similar consequences have been observed regarding the impact of H4K16ac mutants on other biological processes. For example, the *H4-K16Q* substitution significantly reduces lifespan whereas *H4-K16R* shows only a marginal effect 65. Likewise, the frequency of chromosome loss and the levels of rDNA recombination are also higher in *H4-K16Q* strains than in *H4-K16R* mutants 66,67. In line with these observations, our results raise the possibility that the dynamics of H4K16ac, more than only the exact state of such acetylation, is required to regulate the meiotic checkpoint, although the precise mechanism underlying such effect remains to be elucidated.

**Figure 4:**
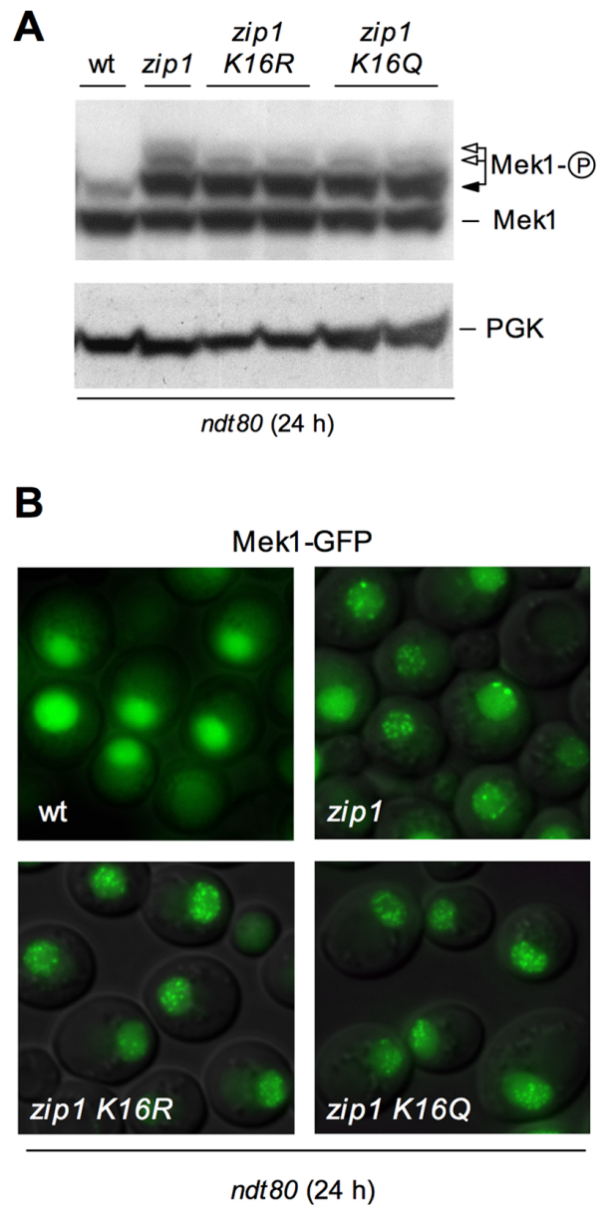
FIGURE 4: Analysis of Mek1 activation and localization in *ndt80*-arrested cells. **(A)** Western blot analysis of different Mek1 phosphorylation forms in *ndt80*-arrested cells after 24 h in meiosis. PGK is shown as a loading control. Strains are DP424 (wild type), DP428 (*zip1*), DP996 (*zip1 H4-K16R*) and DP1002 (*zip1 H4-K16Q*). Two independent clones of DP966 and DP1002 were analyzed. **(B)** Representative images of checkpoint-induced Mek1-GFP foci in wild type (DP584), *zip1* (DP582), *zip1 H4-K16R* (DP1089) and *zip1 H4-K16Q* (DP1090) *ndt80*-arrested cells after 24 h in meiosis.

**Figure 5:**
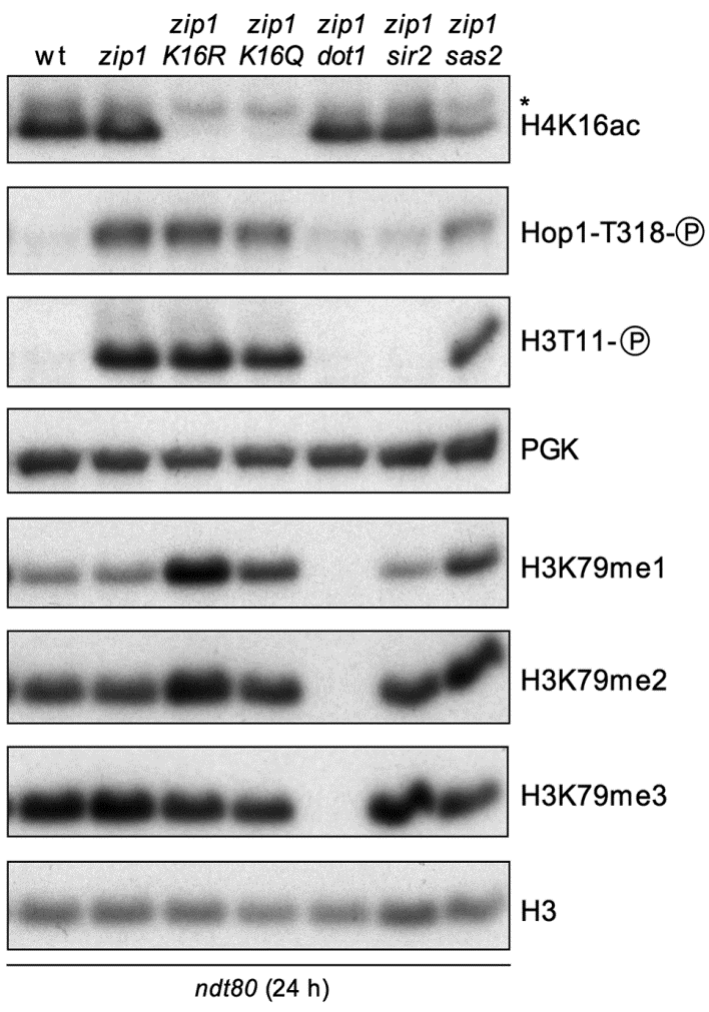
FIGURE 5: The *sir2* mutant, but not *H4-K16Q*, *H4-K16R* or *sas2*, is defective in establishing early markers of checkpoint activation. Western blot analysis of *zip1*-induced Hop1-T318 and H3-T11 phosphorylation, as well as H3K79 methylation, 24 h after meiosis induction in *ndt80*-arrested cells. PGK and total H3 were used as loading controls. Strains are: DP424 (wild type), DP428 (*zip1)*, DP996 (*zip1 H4-K16R*), DP1002 (*zip1 H4-K16Q*), DP655 (*zip1 dot1*), DP1086 (*zip1 sir2*) and DP1073 (*zip1 sas2*).

In principle, the differences observed in Mek1 phosphorylation between *zip1 H4-K16R* and *zip1 H4-K16Q* double mutants and the* zip1* single mutant could be a consequence of their different kinetics in meiotic progression (*zip1* exhibits a profound delay that is bypassed in *zip1 H4-K16R* and *zip1 H4-K16Q*; Figure 2B) or could arise from a direct effect of H4K16 acetylation on Mek1 activation. To distinguish between these two possibilities, we monitored Mek1 phosphorylation in pachytene-arrested *ndt80 *cells. Ndt80 is a meiosis-specific transcription factor required for induction of meiotic middle genes 25,68,69 promoting exit from prophase 70; thus, *ndt80* cells arrest in pachytene independently of the meiotic checkpoint allowing us to analyze the status of checkpoint activation independent of meiotic progression. If H4K16 acetylation were not involved in the establishment of checkpoint-induced Mek1 activation but only in its maintenance, we will expect Mek1 phosphorylation to be similar in *zip1* and in *zip1 H4K16* acetylation mutants, in a *ndt80* background. As shown in Figure 4A, in an *ndt80* background, *zip1 H4-K16R* and *zip1 H4-K16Q* double mutants are only slightly impaired in Mek1 activation. Previous studies have shown that *zip1*-induced checkpoint activation results in different Mek1 phosphorylated forms 21. In Figure 4A we can observe that *H4-K16R* and *H4-K16Q* mutants slightly affected only the upper phosphorylated bands, corresponding to Mek1 autophosphorylation (Figure 4A; white arrows), while the band immediately above the basal form, which depends on Mec1/Tel1 21, remained intact (Figure 4A; black arrow). Moreover, when we analyzed the phosphorylation of H3T11 and Hop1-T318 as additional markers of checkpoint activation in *ndt80* cells, we observed little if any reduction in their phosphorylation levels in the *zip1 H4-K16R* and *zip1 H4-K16Q* mutants when compared to *zip1* (Figure 5). This is in clear contrast with the results of a *zip1 dot1* double mutant in which H3T11ph and Hop1-T318ph were practically abolished (Figure 5), consistent with Dot1 being absolutely required both for checkpoint activation and maintenance 21. These results suggest that the correct acetylation of H4K16 is not required for the establishment of checkpoint-induced Mek1 and Hop1 phosphorylation, but more probably only for its maintenance. If the meiotic prophase block is artificially imposed by means of the *ndt80* mutation, then H4K16ac becomes dispensable to sustain Hop1 and Mek1 activation.

It has been previously demonstrated that, upon meiotic checkpoint activation, the Mek1 effector kinase localizes to discrete nuclear foci that can be detected both on chromosome spreads and in live meiotic cells 12,21. To investigate in more detail the role of H4K16ac in the meiotic checkpoint, we assessed the localization of Mek1-GFP in wild-type, *zip1*, *zip1 H4-K16R* and *zip1 H4-K16Q* cells, always in an *ndt80* background. As expected, *zip1* mutant cells accumulated multiple discrete Mek1-GFP foci during meiotic prophase (Figure 4B) and most *zip1 H4-K16R* and *zip1 H4-K16Q* cells displayed a similar pattern of Mek1 localization (Figure 4B), indicating that formation of *zip1*-induced Mek1 foci is not defective in the absence of normal H4K16ac. This observation suggests that, although H4K16ac is required for sustained meiotic checkpoint activity, it is not necessary for the checkpoint-induced association of Mek1 to meiotic chromosomes.

### The Sir2 and Sas2 proteins are required for proper meiotic checkpoint response

To further investigate the role of H4K16ac in the meiotic recombination checkpoint we studied mutants affecting the metabolism of this residue, such as *sir2* (deficient in a H4K16ac deacetylase), and *sas2* (lacking the main H4K16 acetyltransferase). The relationship of Sir2 with the meiotic checkpoint has been previously reported 36, but a detailed analysis of meiotic progression and checkpoint activity was not described.

**Figure 6:**
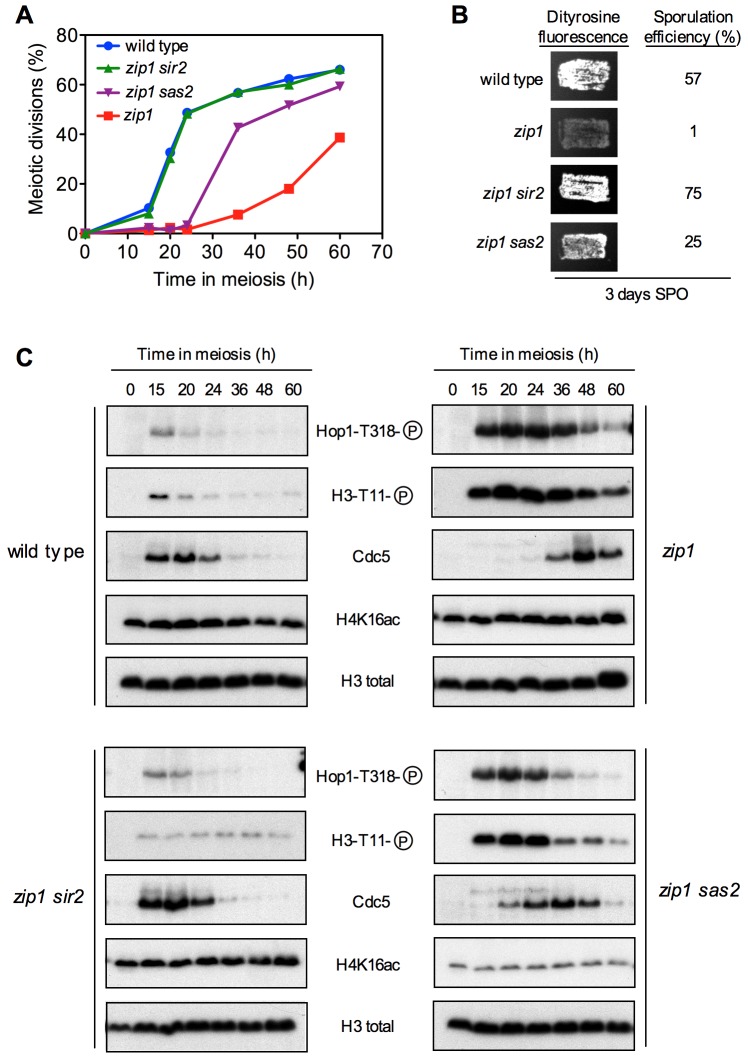
FIGURE 6: The meiotic recombination checkpoint response is impaired in the absence of Sir2 or Sas2. **(A)** Time course of meiotic nuclear divisions; the percentage of cells containing two or more nuclei is represented. **(B) **Dityrosine fluorescence, as a visual indicator of sporulation, and sporulation efficiency, quantified by microscopic examination of at least 300 cells, were examined after 3 days of sporulation on plates. **(C)** Western blot analysis of the indicated proteins during meiosis. Strains are DP421 (wild type), DP422 (*zip1*), DP1401 (*zip1 sir2*) and DP1410 (*zip1 sas2*)*.*

We found that deletion of *SIR2* completely suppressed the meiotic delay imposed by the checkpoint in the *zip1* mutant; that is, the *zip1 sir2* double mutant showed similar kinetics of meiotic progression than the wild type (Figure 6A, S1B) and displayed high levels of sporulation (Figure 6B). Hop1T318 phosphorylation and H3T11 phosphorylation (as a marker of Mek1 activity) were drastically reduced in *zip1 sir2* compared to *zip1* and, according with the meiotic progression, Cdc5 production was restored to wild-type kinetics in *zip1 sir2* (Figure 6C). Disruption of *SAS2* also alleviated the *zip1* meiotic block, but to a lesser extent than *zip1 sir2* did (Figure 6A, 6B, S1B). Consistent with this intermediate effect, Hop1T318 and H3T11 phosphorylation showed a moderate reduction, and Cdc5 dynamics was only partially restored in *zip1 sas2* (Figure 6C). Thus, in *NDT80+* cells competent for meiotic progression, the checkpoint phenotype resulting from the lack of the Sir2 deacetylase is similar to that produced by the *H4-K16Q* mutation mimicking constitutive acetylation, and the effect produced by the absence of the Sas2 acetyltransferase parallels that of the *H4-K16R* mutation preventing acetylation of this residue (Figures 2 and 3).

The checkpoint impact of *SIR2* and *SAS2* deletions was also analyzed in *ndt80* mutant cells by monitoring the levels of *zip1*-induced Hop1T318 and H3T11 phosphorylation. In the case of *sir2*, we found a complete abrogation of both phosphorylation events (Figure 5), indicating that, in contrast to *H4-K16Q*, the *sir2 *mutant is defective both in the establishment and maintenance of the meiotic checkpoint, in a similar way to *dot1*. The fact that the lack of the H4K16ac Sir2 deacetylase does not cause exactly the same effect as the mimicked constitutive acetylation of the *H4-K16Q* mutant in *ndt80* strains suggests that Sir2 has additional checkpoint functions. On the other hand, in *ndt80* cells, *SAS2* disruption only showed a marginal effect on both H3T11 and Hop1 phosphorylation, similar to what we observed with the acetylation-defective *H4-K16R* mutant (Figure 5), indicating that Sas2 is primarily involved in checkpoint maintenance.

We also monitored the state of H4K16ac and, as we showed above (Figure 1), it was also unaffected when the checkpoint was triggered by *zip1* in *ndt80*-arrested cells (Figure 5). Strikingly, we found that the disruption of *SIR2* did not significantly increase global levels of H4K16ac in either *NDT80* or *ndt80* cells (Figures 5 and 6C), consistent with the notion that Sir2 is not the main genome-wide H4K16ac deacetylase and its action may be specifically restricted to precise heterochromatic domains 53,54. On the other hand, *SAS2* deletion clearly, but not completely, reduced H4K16ac (Figures 5 and 6C), suggesting that Sas2 is the main, but not the only, H4K16 acetyltransferase acting in the meiotic cell cycle.

### Cross-talk between H4K16 acetylation and H3K79 methylation

**Figure 7:**
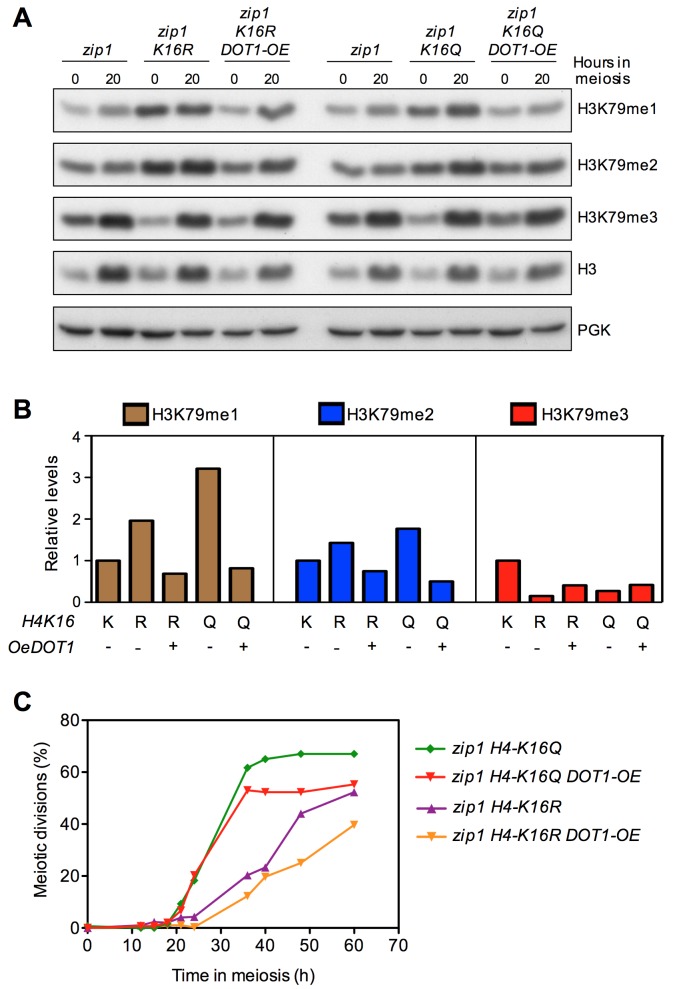
FIGURE 7: *DOT1* overexpression partially restores the meiotic checkpoint in H4K16ac-deficient mutants. **(A)** Western blot analysis of H3K79 methylation species in vegetative (T=0h) and meiotic cells (T=20h). Total H3 and PGK were used as loading controls. **(B)** Quantification of relative levels of the H3K79 methylation forms at T=0 from the blots shown in (A). Total H3 signal was used for normalization. **(C)** Time course of meiotic nuclear divisions; the percentage of cells with two or more nuclei is presented. Strains are DP422 (*zip1)*, DP995 (*zip1 H4-K16R*) and DP1001 (*zip1 H4-K16Q*), transformed either with an empty vector or with the high-copy pSS63 *DOT1* overexpression plasmid (*DOT1-OE*).

Previous studies have shown that some histone PTMs positively or negatively affect other histone marks in what has been described as histone cross-talk, adding an extra layer of complexity to the control of different chromatin processes 71,72,73. One example is the tri-methylation of H3K79 by Dot1, which is completely dependent upon the prior ubiquitylation of H2BK123 by Rad6/Bre1 74. It has also been described that H4K16ac modulates Dot1-dependent H3K79 methylation by promoting Dot1 binding to a short basic patch in the histone H4 tail in competition with Sir3 75. Since Dot1-dependent H3K79 methylation is required for the meiotic recombination checkpoint 21,37 it was possible that the impact of H4K16ac on the checkpoint (Figures 2 and 3) was exerted via regulation of H3K79me. To explore this possibility, we first analyzed the effect of *H4-K16R* and *H4-K16Q *mutations on H3K79 mono-, di- and tri-methylation in *zip1 ndt80* checkpoint-activated and pachytene-arrested cells (Figures 5 and 7A, 7B). Given the distributive mode of action of the Dot1 methyltransferase 76, an impaired Dot1 catalytic activity is manifested as a reduction in H3K79me3 concomitant with an increase in H3K79me1 and H3K79me2 21,76. Indeed, we observed higher levels of H3K79me1 and H3K79me2 in both H4K16ac-defective mutants, as well as a reduction in those of H3K79me3 (Figures 5 and 7A, 7B), which is the most relevant form to sustain the meiotic checkpoint response 21. Thus, these findings suggest that H4K16ac mutants affect the activity of Dot1. We also observed that, like *H4-K16R*, the absence of the H4K16 acetyltransferase Sas2 also increased H3K79me1 and H3K79me2 and reduced H3K79me3 (Figure 5). Curiously, disruption of *SIR2*, did not have any effect on global H3K79me levels (Figure 5), again consistent with the notion that Sir2 meiotic checkpoint function can be exerted, at least in part, in a way that is independent from a global activity on H4K16ac.

Since Dot1 catalytic activity appears to be compromised in H4K16ac mutants, we explored whether *DOT1* overexpression would restore normal H3K79me3 levels and meiotic checkpoint function in *zip1 H4-K16R* or *zip1 H4-K16Q* double mutants. *DOT1* was overexpressed from a high-copy plasmid (Figure S2) and the pattern of H3K79me was analyzed at 0 h and 20 h after meiotic induction (Figure 7A, 7B). We found that the increased H3K79me1 and H3K79me2 levels observed in the *zip1 H4-K16R* and *zip1 H4-K16Q* mutants were reduced upon *DOT1* overexpression (Figure 7A, 7B). On the contrary, high doses of Dot1 increased the amount of H3K79me3 in *zip1 H4-K16R* and *zip1 H4-K16Q*, although it did not reach normal wild-type levels (Figure 7A, 7B). These observations confirm that overexpression of *DOT1* can partially compensate for the crippled Dot1 methyltransferase activity when H4K16ac metabolism is altered; therefore, we analyzed the effect on the meiotic checkpoint by monitoring the kinetics of meiotic divisions.

We have shown before that *H4-K16R* releases the checkpoint-dependent *zip1* meiotic delay to some extent and *H4-K16Q* completely alleviates the *zip1* block (Figure 2B). Interestingly, *DOT1* overexpression resulted in less efficient meiotic progression in *zip1 H4-K16R* and *zip1 H4-K16Q *cells compared to the controls transformed with empty vector (Figure 7C, S1C), consistent with a partial restoration of the checkpoint. Altogether, these results suggest that the effect of *H4-K16R* and *H4-K16Q* mutations on the meiotic checkpoint triggered by a *zip1* mutant is exerted, at least in part, through their effect on modulating proper H3K79 methylation pattern.

### Relationship between Sir2, H4K16ac and Pch2 nucleolar localization

In *Saccharomyces cerevisiae*, the Pch2 protein is a negative regulator of Hop1 chromosomal abundance in synapsed chromosomes 77,78, but it is required for the *zip1*-induced meiotic checkpoint promoting Hop1 phosphorylation at T318 36,59. The majority of Pch2 localizes to the unsynapsed nucleolar region of chromosome XII that contains the ribosomal RNA genes (rDNA), where it is required to exclude the meiosis-specific Hop1 protein from the nucleolus. This nucleolar localization of Pch2 is completely dependent on the Sir2 deacetylase, which is also located in the rDNA 36, and deletion of *SIR2* impairs the meiotic checkpoint (Figures 5 and 6). Moreover, the Dot1 meiotic checkpoint factor regulates both Sir2 and Pch2 nucleolar localization 21,37. This scenario points to a pivotal role for the nucleolar Pch2 in the pachytene checkpoint 59 and prompted us to investigate if *H4-K16R* and/or *H4-K16Q* mutations affected the nucleolar localization of Pch2 on meiotic chromosome spreads.

**Figure 8:**
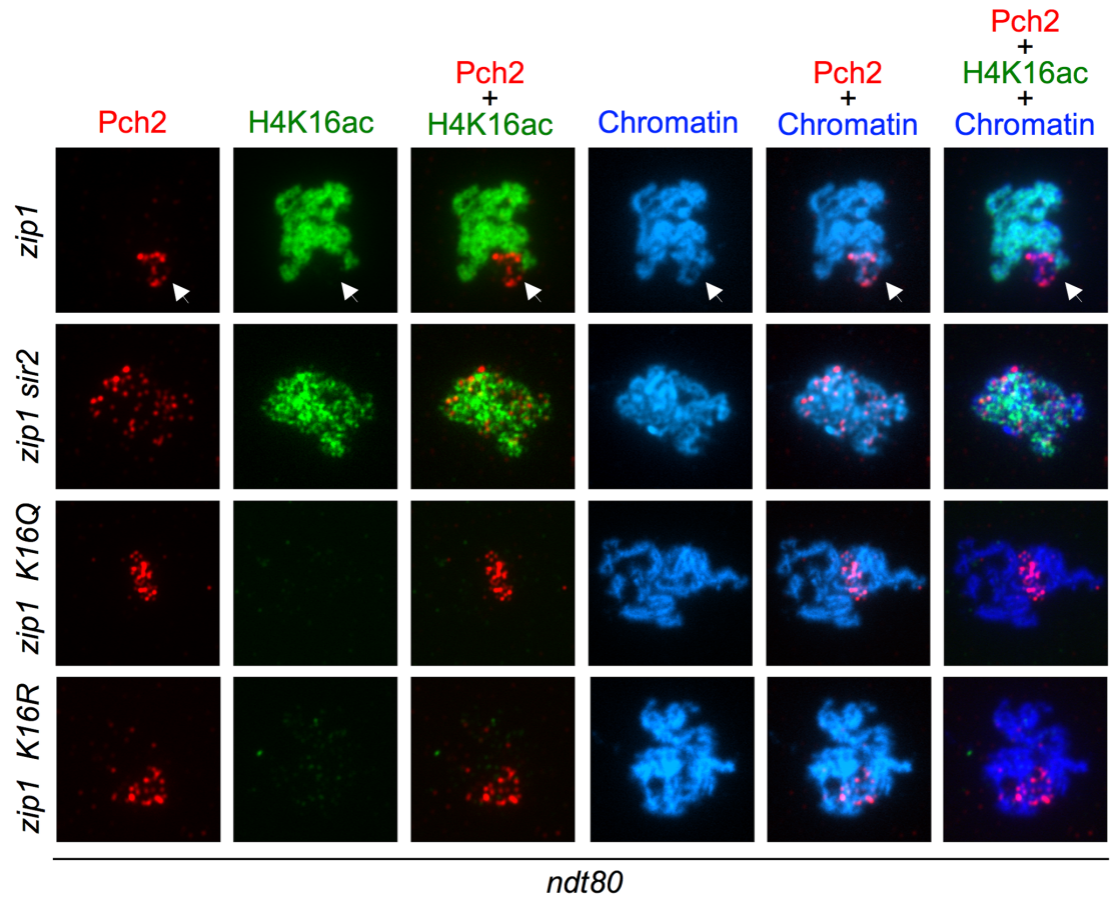
FIGURE 8: Analysis of Pch2 localization in H4K16ac-deficient mutants. Immunofluorescence of meiotic chromosome spreads from *zip1* (DP1123), *zip1 sir2* (DP1124), *zip1 H4-K16R* (DP1121) and *zip1 H4-K16Q* (DP1139) stained with DAPI (blue) as well as with anti-HA to detect Pch2-HA (red) and anti-H4K16ac (green) antibodies. The arrows point to the rDNA region where Pch2 accumulates and is devoid of H4K16ac. Representative nuclei are shown. Spreads were prepared after 24 h of meiotic induction in *ndt80* strains.

In *zip1* cells, when the meiotic checkpoint is activated, Pch2 localization is limited to the nucleolar (rDNA) region (Figure 8). As it has been previously shown 36, in *zip1 sir2* cells the nucleolar concentration of Pch2 was lost and the protein appeared in form of foci dispersed throughout the meiotic chromosomes (Figure 8). Then, we analyzed Pch2 distribution in the *zip1 H4-K16Q* and *zip1 H4-K16R* mutants. We found that the Pch2 signal was still located in a restricted chromosomal area, presumably the rDNA region, but it was somehow more diffused although to a lesser extent than in *zip1 sir2* (Figure 8). Thus, like Dot1 and Sir2, these findings point to a role for H4K16ac in delimiting the nucleolar confinement of Pch2 and its exclusion from the rest of the chromatin, although the action of Sir2 must not be exerted only on H4K16ac because the effect of *SIR2* deletion on Pch2 localization is stronger than that of H4K16 mutations.

**Figure 9:**
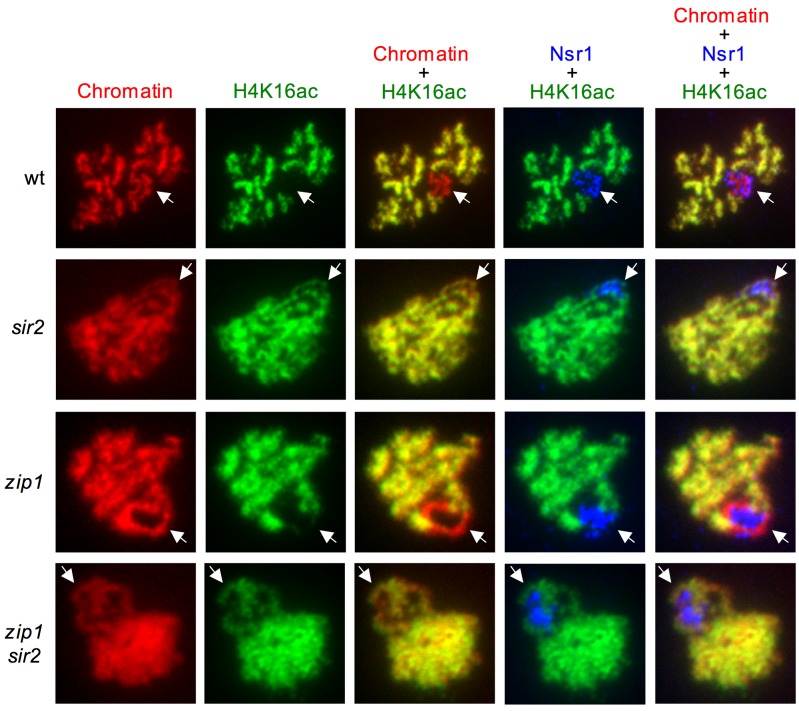
FIGURE 9: Sir2 excludes H4K16ac from the rDNA region. Immunofluorescence of meiotic chromosome spreads from wild type (BR2495), *sir2* (DP262), *zip1* (DP1123) and *zip1 sir2* (DP1124), stained with DAPI (red) as well as with anti-H4K16ac (green) and anti-Nsr1 (blue) antibodies. The arrows point to the rDNA region identified by Nsr1 staining. Representative nuclei are shown. Spreads were prepared after 16 h of meiotic induction for BR2495 and DP262 and 24 h for DP1123 and DP1124.

In addition, we also examined the distribution of H4K16ac on meiotic chromosomes. We used an antibody that recognizes the nucleolar Nsr1 protein involved in ribosome biogenesis 79 to unambiguously identify the rDNA region, which often appears as a chromatin loop on preparations of spread meiotic chromosomes. Strikingly, the H4K16ac histone mark was completely excluded from the rDNA in both wild-type and *zip1* nuclei (Figure 9), also displaying an exclusive localization pattern with that of nucleolar Pch2 (Figure 8, arrow). However, in the absence of Sir2, H4K16ac was distributed all along the chromatin, showing a complete co-localization with the DAPI staining, including the rDNA region marked by the nucleolar Nsr1 protein (Figure 9). Consistent with microarray studies in vegetative cells 53 and with our western blot analysis of global meiotic levels of H4K16ac (Figures 5 and 6), the H4K16ac signal on the bulk of the genome was not significantly altered in *sir2* mutants (Figure 9). These results indicate that Sir2 is the major deacetylase specifically responsible for preventing H4K16 acetylation in the rDNA during meiosis.

Besides the impact on the meiotic recombination checkpoint, it has been shown that *SIR2* disruption significantly alters the genomic distribution of Spo11-induced DSBs; with some genes displaying increased levels of DSBs whereas others experience reduced levels of DSBs in the absence of Sir2 80. Two defined genomic domains, such as subtelomeric regions and the rDNA array, show elevated recombination in the *sir2* mutant 80. Pch2 also prevents recombination at the rDNA by excluding Hop1 from the nucleolar region 36,59. Moreover, Sir2 and Pch2 modulate the protection of DSB-induced meiotic instability at the rDNA borders 81. It has been proposed that the effect of Sir2 on recombination at subtelomeric regions is exerted through the regulation of H4K16ac; however, the heterogeneous effect of Sir2 on the global meiotic DSB landscape implies that multiple factors and targets must be involved in addition to H4K16ac 80.

### Concluding remarks

**Figure 10:**
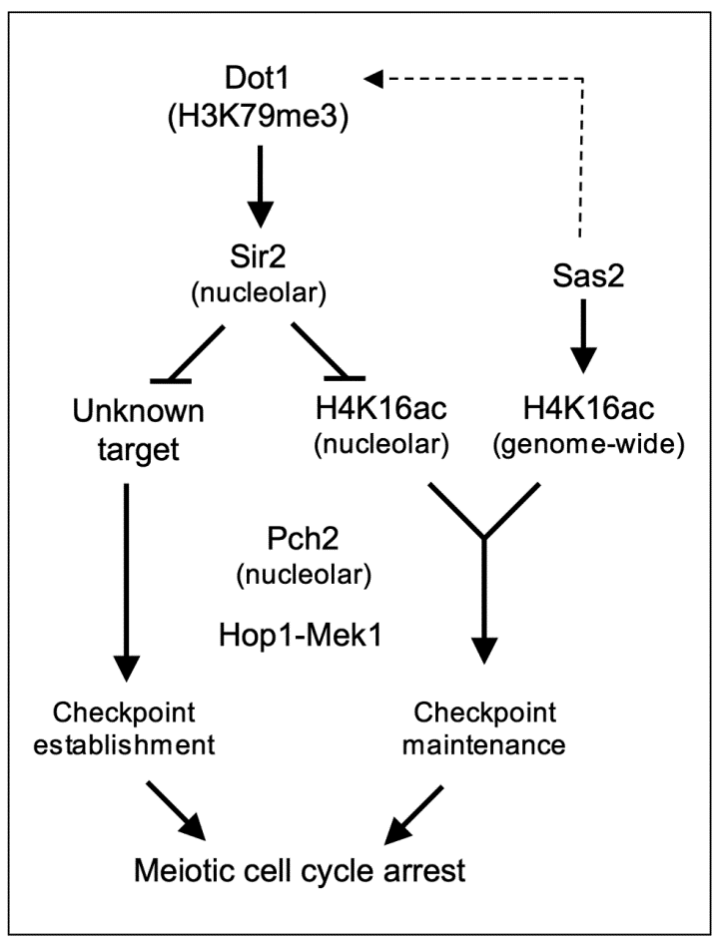
FIGURE 10: A model for the regulation of the meiotic checkpoint by histone post-translational modifications. See text for details.

In this work we have explored the functional contribution of H4K16ac, the Sir2 deacetylase and the Sas2 acetyltransferase in the meiotic recombination checkpoint triggered by synaptonemal complex defects. In line with previous observations, our results indicate that an intricate network of histone PTMs fine-tune this meiotic quality control mechanism (Figure 10). We propose that reduced levels of Dot1-mediated H3K79me3 at the rDNA enable the enrichment of Sir2 in the nucleolus. The presence of Sir2 at the rDNA region is responsible for the low level of H4K16ac in this area and, together with an additional unknown Sir2 target, confines Pch2 in the nucleolus. The Pch2 ATPase is critical to orchestrate the proper balance between the amount of Hop1 bound to chromosome axes and phosphorylated Hop1, which in turn sustains Mek1 activation 21,59. Nevertheless, the precise mechanism by which nucleolar Pch2 regulates the phosphorylation status of the Hop1 checkpoint adaptor located at the axes and excluded from the rDNA remains to be determined.

Curiously, when meiotic progression is prevented by the *ndt80* mutation, we have observed different checkpoint activity phenotypes resulting from deletion of *SIR2* compared with *H4-K16* or *sas2* mutants. Whereas Sir2 is required for Mek1 activation in any condition, Sas2/H4K16ac only affect the maintenance of Mek1 activation in *NDT80*-proficient cells, thus supporting the notion that Sir2 acts on additional targets.

We hypothesize that the general status of H4K16ac modulates DNA repair pathways involved in the resolution of recombination intermediates accumulated in *zip1* triggering the checkpoint arrest. Alteration of H4K16ac dynamics by *SAS2* deletion or *H4-K16* mutations, would allow the Ndt80-dependent repair of those intermediates thus allowing meiotic progression in *zip1.* Further experimental work will be required to explore this possibility.

## Materials and Methods

### Yeast strains

Yeast strains genotypes are listed in Table S1. All the strains are in the BR1919 or BR2495 genetic background 82. Gene deletion and tagging were performed using a PCR-based approach or by genetic crosses always in an isogenic background. The *dot1::URA3*, *zip1::LYS2, zip1::LEU2, sir2::URA3* and *ndt80::LEU2* deletions were previously described 5,26,31,37,83. In the plasmid-borne *H4-K16R and H4-K16Q* mutants, both genomic copies of the histone H3-H4 encoding genes (*HHT1-HHF1* and *HHT2-HHF2*) were deleted and the wild-type *HHT2-HHF2* genes or the modified *HHT2-hhf2(K16R)* or *HHT2-hhf2(K16Q) *versions were expressed from the centromeric plasmids pRM204, pWD23 and pWD25, respectively, as the only source of H3-H4 histones 65. Alternatively, both copies of the histone H4-encoding genes *HHF1* and *HHF2* were mutated in their genomic loci to *K16R* and *K16Q* following the *delitto perfetto *approach 84. N-terminal tagging of Pch2 with three copies of the HA epitope and the *MEK1-GFP* construct were previously described 21,36. *DOT1-HA* was overexpressed from the pSS63 plasmid 37.

### Meiotic time courses

Strains were grown on 2xSC (3,5 ml) for 20-24 h and then transferred to 2,5 ml of YPDA where they were incubated to saturation for an additional 8 h. Cells were then harvested, washed with 2% potassium acetate (KAc), resuspended into 10 ml of KAc and incubated at 30°C with vigorous shaking (235 rpm) to induce meiosis and sporulation. 20 mM adenine and 10 mM uracil was added to both YPDA and KAc media. Culture volumes were scaled up when needed. Aliquots of cells were removed at different time points for analysis. To analyze meiotic divisions, cells were fixed in 70% ethanol, washed in phosphate-buffered saline (PBS) and stained with 1 mg/ml DAPI for 15 min at room temperature. Nuclei were observed by fluorescence microscopy and at least 300 cells were scored for each strain at each time point in every experiment. Meiotic kinetics experiments were repeated several times and representative experiments are shown. Dityrosine fluorescence was analyzed as previously described 37 and spore viability was determined by tetrad dissection.

### Western blotting

TCA yeast whole cell extracts from 5-10 ml aliquots of meiotic cultures were prepared as described previously 18 and proteins were resolved by SDS-PAGE and then transferred to PVDF membranes. To resolve the phosphorylated forms of Mek1, 10% SDS-PAGE gels with a 29:1 ratio of acrylamide:bisacrylamide containing 37,5 μM Phos-tag reagent (Wako) and 75 μM MnCl_2_ were prepared as described 18,21, whereas normal 15% or 10% gels (acrylamide:bisacrylamide 37,5:1) were used for detection of H4K16ac, H3T11ph and H3K79me or Mek1, Hop1-T318ph, Cdc5 and Dot1-HA, respectively. Blots were probed with the following primary antibodies: rabbit polyclonal antibodies raised against Mek1 (1:1000) 13, Hop1-T318 (1:1000; kindly provided by J. Carballo), H3T11ph (1:2000; Abcam 5168), H4K16ac (1:2000; Millipore 07-329), H3K79-me1 (1:1000; Abcam ab2886), H3K79-me2 (1:2000; Abcam ab3594) and H3K79-me3 (1:2000; Abcam ab2621); goat polyclonal antibody against Cdc5 (1:1000; Santa Cruz Biotechnology sc-6733); mouse monoclonal antibody against the HA epitope (1:2000; Roche 12CA5). A mouse monoclonal antibody directed against 3-phosphoglycerate kinase (PGK) (1:10000; Molecular Probes A-6457) or a rabbit polyclonal antibody against histone H3 (1:5000; Abcam ab1791) were used as loading controls. HRP-conjugated secondary antibodies were from GE Healthcare (NA934 and NA931) or Santa Cruz Biotechnology (sc-2033). The Pierce ECL or ECL-2 reagents (Thermo Scientific) were used for detection and the signal was captured on film (Amersham Hyperfilm ECL; GE Healthcare) and/or with a ChemiDoc XRS (BioRad) system, using the Quantity One software (Bio-Rad). The same software was used to quantify protein levels.

### Cytology

Whole cell images were captured with a Nikon Eclipse 90i fluorescence microscope controlled with the MetaMorph software (Molecular Devices) and equipped with an Orca-AG (Hamamatsu) CCD camera and a PlanApo VC 100X 1.4 NA objective. To analyze Mek1-GFP foci in live meiotic cells, exposure time was 1 second and stacks of 11 planes at 0,4 μm were captured. Maximum intensity projections were generated with the NIH ImageJ software (http://rsb.info.nih.gov/ij ). To outline the contour of the cells in the representative whole-cell images presented, an overlay of the DIC image with 15-20% transparency over the GFP signal is shown. Immunofluorescence of meiotic chromosome spreads was performed as previously described 36. To detect the HA-tagged Pch2 and H4K16ac, a mouse monoclonal anti-HA antibody (12CA5, Roche) or a rabbit polyclonal anti H4K16ac (Millipore 07-329) were used at 1:200 dilution. Nsr1 was detected with a mouse monoclonal antibody (clone 31C4, ThermoFisher MA1-10030) used at 1:200 dilution. Alexa-Fluor-488 and Alexa-Fluor-594-conjugated secondary antibodies from Molecular Probes were used at 1:200 dilution. Images were captured with the same equipment as indicated above.

## SUPPLEMENTAL MATERIAL

Click here for supplemental data file.

All supplemental data for this article are also available online at http://microbialcell.com/researcharticles/impact-of-histone-h4k16-acetylation-on-the-meiotic-recombination-checkpoint-in-saccharomyces-cerevisiae/.
