# Supplementary material for: Impact of histone H4K16 acetylation on the meiotic recombination checkpoint in Saccharomyces cerevisiae
Source: Microb Cell. 2016 Dec 4;3(12):606–20. doi: 10.15698/mic2016.12.548 (PMC5348980; doi:10.15698/mic2016.12.548)
Supplement: Supplementary file 1 [file mic-03-606-s01.pdf]

SUPPLEMENTAL INFORMATION

Impact of histone H4K16 acetylation on the meiotic recombination checkpoint in *Saccharomyces cerevisiae*

Santiago Cavero[1, 2], Esther Herruzo[1], David Ontoso[1, 3] and Pedro A. San-Segundo[1, *]

[1] Instituto de Biología Funcional y Genómica. Consejo Superior de Investigaciones Científicas and University of Salamanca, 37007 Salamanca, Spain

[*] To whom correspondence should be addressed: Email: pedross@usal.es

Table S1. *Saccharomyces cerevisiae* strains

Strain	Genotype *
BR1919-2N	*MAT***a**/*MAT*α *leu2-3,112 his4-260 ura3-1 ade2-1 thr1-4 trp1-289*
BR2495	*MAT***a**/*MAT*α *leu2-27/leu2-3,112 his4-280/his4-260 trp1-1/trp1-289 arg4-8/ARG4 thr1-1/thr1-4 cyh10/CYH10 ura3-1 ade2-1*
DP262	BR2495 *sir2::URA3 PCH2-3HA*
DP421	BR1919-2N *lys2ΔNheI*
DP422	DP421 *zip1::LYS2*
DP424	DP421 *ndt80::LEU2*
DP428	DP421 *zip1::LYS2 ndt80::LEU2*
DP582	DP421 *zip1::LYS2 ndt80::LEU2 MEK1-GFP::kanMX6*
DP584	DP421 *ndt80::LEU2 MEK1-GFP::kanMX6*
DP634	DP421 *(hht1-hhf1)::kanMX6 (hht2-hhf2)::natMX4 p(HHT2-HHF2)::TRP1*
DP635	DP421 *(hht1-hhf1)::kanMX6 (hht2-hhf2)::natMX4 p(HHT2-hhf2-K16R)::TRP1*
DP636	DP421 *(hht1-hhf1)::kanMX6 (hht2-hhf2)::natMX4 p(HHT2-hhf2-K16Q)::TRP1*
DP639	DP421 *(hht1-hhf1)::kanMX6 (hht2-hhf2)::natMX4 p(HHT2-HHF2)::TRP1 zip1::LYS2*
DP640	DP421 *(hht1-hhf1)::kanMX6 (hht2-hhf2)::natMX4 p(HHT2-hhf2-K16R)::TRP1 zip1::LYS2*
DP641	DP421 *(hht1-hhf1)::kanMX6 (hht2-hhf2)::natMX4 p(HHT2-hhf2-K16Q)::TRP1 zip1::LYS2*
DP655	DP421 *zip1::LYS2 ndt80::LEU2 dot1::kanMX6*
DP994	DP421 *hhf1-K16R hhf2-K16R*
DP995	DP421 *hhf1-K16R hhf2-K16R zip1::LYS2*
DP996	DP421 *hhf1-K16R hhf2-K16R zip1::LYS2 ndt80::LEU2*
DP1000	DP421 *hhf1-K16Q hhf2-K16Q*
DP1001	DP421 *hhf1-K16Q hhf2-K16Q zip1::LYS2*
DP1002	DP421 *hhf1-K16Q hhf2-K16Q zip1::LYS2 ndt80::LEU2*
DP1073	DP421 *zip1::LYS2 ndt80::LEU2 sas2::natMX4*
DP1086	DP421 *zip1::LYS2 ndt80::LEU2 sir2::URA3*
DP1089	DP421 *zip1::LYS2 ndt80::LEU2 hhf1-K16R hhf2-K16R MEK1-GFP::kanMX6*
DP1090	DP421 *zip1::LYS2 ndt80::LEU2 hhf1-K16Q hhf2-K16Q MEK1-GFP::kanMX6*
DP1121	DP421 *zip1::LEU2 ndt80::kanMX6 hhf1-K16R hhf2-K16R PCH2-3HA*
DP1123	DP421 *zip1::LEU2 ndt80::kanMX6 PCH2-3HA*
DP1124	DP421 *zip1::LEU2 ndt80::kanMX6 sir2::URA3 PCH2-3HA*
DP1139	DP421 *zip1::LEU2 ndt80::kanMX6 hhf1-K16Q hhf2-K16Q PCH2-3HA*
DP1401	DP421 *zip1::LYS2 sir2::URA3*
DP1410	DP421 *zip1::LYS2 sas2::natMX4*

* Unless indicated, all diploid strains are homozygous for the markers.

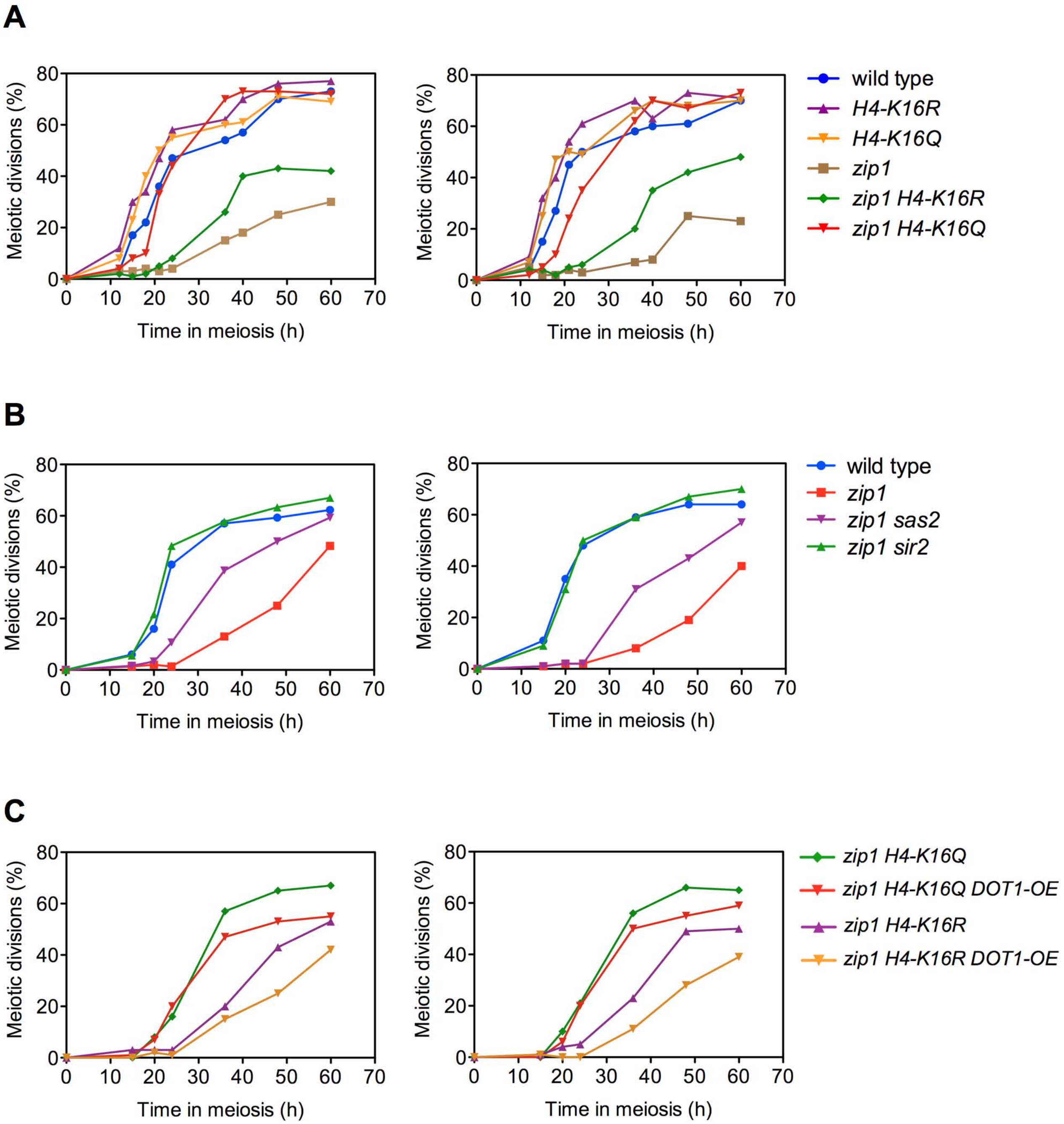

Figure S1. Additional replicates of the representative meiotic time courses shown in main figures

(A) Two replicates of the time course shown in Figure 2B

(B) Two replicates of the time course shown in Figure 6A

(C) Two replicates of the time course shown in Figure 7C

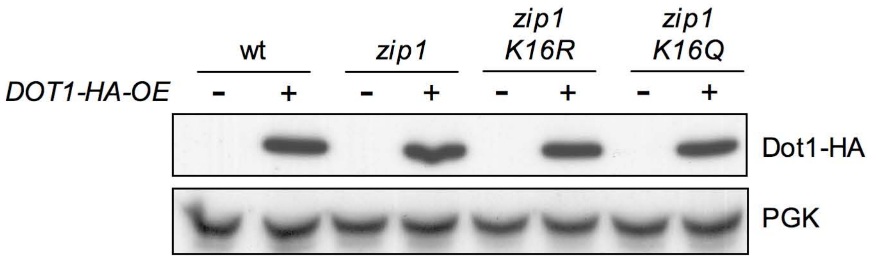

Figure S2. *DOT1* overexpression in H4K16ac mutants
Western blot analysis of *DOT1* overexpression in wild-type (DP421), *zip1* (DP422), *zip1 H4-K16R* (DP995) and *zip1 H4-K16Q* (DP1001) strains transformed either with an empty vector or with a high-copy plasmid expressing *DOT1-HA* (pSS63). PGK was used as a loading control.
